# The Length Change Ratio of Ground Granulated Blast Furnace Slag-Based Geopolymer Blended with Magnesium Oxide Cured in Various Environments

**DOI:** 10.3390/polym14163386

**Published:** 2022-08-18

**Authors:** Yen-Chun Chen, Wei-Hao Lee, Ta-Wui Cheng, Walter Chen, Yeou-Fong Li

**Affiliations:** 1 Institute of Mineral Resources Engineering, National Taipei University of Technology, 1, Sec. 3, Chung-Hsiao E. Rd., Taipei 10608, Taiwan; 2 Department of Civil Engineering, National Taipei University of Technology, Taipei 10608, Taiwan

**Keywords:** geopolymer, magnesium oxide, shrinkage, length change ratio, curing environment

## Abstract

Geopolymer (GP) has been considered a potential material to replace ordinary Portland cement (OPC) because of its excellent mechanical properties and environmentally friendly process. However, the promotion of GP is limited due to the large shrinkage and the different operating procedures compared to cement. This study aims to reduce the shrinkage of ground granulated blast furnace slag (GGBFS) based GP by the hydration expansion properties of activated magnesium oxide (MgO). The slurry of GP was blended from GGBFS, MgO, and activator; and the compositions of the activator are sodium hydroxide (NaOH), sodium silicate (Na_2_SiO_3_), and alumina silicate(NaAlO_2_). Herein, the GGFBS and MgO were a binder and a shrinkage compensation agent of GP, respectively. After unmolding, the GP specimens were cured under four types of environments and the lengths of the specimens were measured at different time intervals to understand the length change ratio of GP. In this study, two groups of GP specimens were made by fixing the activator to binder (A/B) ratio and the fluidity. The test results show that adding MgO will reduce the shrinkage of GP as A/B ratio was fixed. However, fixing the fluidity exhibited the opposite results. The X-ray diffraction (XRD) was used to check the Mg(OH)_2_ that occurred due to the MgO hydration under four curing conditions. Three statistical and machine learning methods were used to analyze the length change of GP based on the test data. The testing and analysis results show that the influence of curing environments is more significant for improving the shrinkage of GP than additive MgO.

## 1. Introduction

Reinforced concrete has been widely used in construction due to its low cost and easy to apply. The carbon dioxide (CO_2_) emission from the production of ordinary Portland cement (OPC) is 700~1000 kg/ton, with the total accounting for approximately 5% of global CO_2_ emissions [1,2,3,4]. The manufacturing process of OPC also releases pollutants, such as sulfur dioxide, nitrogen oxides, and carbon monoxide [5]. To slow down global warming, the cement industry has focused on reducing the carbon footprint of cement, mainly through alternative energy sources, alternative materials, and recycled cement [6,7,8,9].

Geopolymer (GP) is an alkali-activated material (AAM), the structure of which is an amorphous or semi-crystalline material similar to zeolite [10,11,12,13,14,15]. GP has been considered sustainable material due to the lower CO_2_ emission than OPC, however, the actual reduction in CO_2_ emissions remains to be confirmed due to the factors of carbon footprint such as raw materials and transportation [9,16,17,18]. Compared with OPC, GP also has better mechanical properties, and resistance to fire, sulfate, and chloride-induced corrosion. Therefore, there are many studies and applications focused on GP issue in recent years, for example, recycled waste can produce bricks, fire-proof materials, and other productions [19,20,21,22,23,24,25,26,27,28,29,30,31,32].

However, GP also has limitations of application; the process of geopolymerization will dehydrate and cause excessive shrinkage and cracks [33,34]. The drying shrinkage of GP has been considered to be caused by the higher porosity and smaller diameter pore size distribution (less than 25 nm) [35,36]. The shrinkage of GGBS-based GP mortar has been tested and it is up to six times the shrinkage of OPC. Using sodium carbonate as an alkaline activator can reduce its shrinkage, but can also reduce its compressive strength [37].

The studies indicate that the GP is sensitive to the curing environment. The greater shrinkage and lower strength occur when GP is cured at a lower relative humidity environment, while it can be improved as curing in water [35,38]. The drying shrinkage of the GGBFS-based GP has strongly dependent on the relative humidity and the cations. The cations may change the structure of C-A-S-H and make it easier to shrink in a dry environment [39]. In the drying shrinkage process of metakaolin (MK)-based GP, it is considered that the slurry loses free water from macro-pores first without capillary pressure. Then, the constitution water starts to lose and cause shrinkage [40].

Adding filler and fiber are the most popular methods to improve the shrinkage of GP, such as sand, wollastonite, and glass fibers. The GGBFS-based and MK-based GP with fillers can reduce the shrinkage by 27% to 77%. Fillers can reduce the endogenous shrinkage because the granular particle and fiber skeleton change the pore size distribution so it might reduce the tensile forces [41]. It has also been found that increasing the sodium hydroxide (NaOH) concentration of the activator and adding NaAlO_2_ can reduce shrinkage [41,42].

The additive expansion materials, such as MgO, FGD-gypsum, dolomite, etc., and shrinkage-reducing admixture (SRA) have also been studied to reduce the shrinkage of GP. The shrinkage is decreased due to the volume expansion caused by the hydration of MgO to Mg(OH)_2_ [43]. When FGD-gypsum and dolomite are activated with NaOH solution to form CaCO_3_ and Na_2_CO_3_ to compensate for shrinkage. Nevertheless, when the addition of the expansion materials was used, the greater the water content, the larger the drying shrinkage [44]. The mechanism of SRA is declining the surface tension of the pore and capillary stress because SRA can modify the pore structure and increase the pore size [45].

Due to the complicated mechanisms of OPC and GP, the artificial neural network (ANN), machine learning (ML), and multiple linear regression (MLR) have been widely used to predict the compressive strength and shrinkage behavior of OPC concrete; and some studies have started to develop the ANN and ML to predict the compressive strength of GP [46,47,48,49,50,51,52,53]. For shrinkage of GP, a study indicates that the accuracy of the ANN model is higher than that of the MLR [54].

In this study, the activator molarity, unmolding time, MgO content, and curing conditions were used to investigate the shrinkage of GGBFS-based GP. Besides, three kinds of the ML, including random forest, discission tree, and the MLR, were used to analyze the 28 days’ length change ratio of GP to evaluate the effect of the above parameters.

## 2. Material

The purpose of this study was to add activated MgO into the GGBFS-based GP to compensate for the shrinkage of GP. In this work, GP was made by GGBFS activated with sodium-based alkaline activators which contain sodium hydroxide (NaOH), sodium silicate (Na_2_SiO_3_), and sodium aluminate (NaAlO_2_). To facilitate the comparison of the length change ratio, this study did not use aggregates in the experiment to avoid the influence of aggregates and their moisture content.

### 2.1. GGBFS

The GGBFS powder was produced by CHC Resources Corp. (Kaohsiung, Taiwan), with a mean particle size (D_50_) is 12.33 μm, specific surface area of 4000 cm^2^/g, and bulk density of 2.9. The chemical composition of GGBFS is shown in Table 1.

### 2.2. Alkaline Activator

The sodium-based alkaline activator was used in this study; the activator contains tap water, NaOH (industrial-grade, 95%), Na_2_SiO_3_ (Baumé scale is 15°, Na_2_O content is 9–10%, and SiO_2_ content is 28–30%), and NaAlO_2_ (molar ratio of Na/Al is 1.18, Na_2_O is 19.5%, Al_2_O_3_ is 26.5%). The alkaline activators with the molarity of NaOH include 2 M, 4 M, 6 M, and 8 M. The molar ratio of Si/Na and Si/Al is 1.28 and 50, respectively.

### 2.3. MgO

The type of MgO used in this study was UC95S produced by Ube Material Industries LTD, Japan. The mean particle size (D_50_) and specific surface area of MgO are 2.735 μm and 25.0 m^2^/g. The chemical composition of MgO is shown in Table 2. The activity of MgO was 78.0% tested by the hydration method according to YB/T 4019–2006.

## 3. Experiment

The preparation of the specimen, experimental planning, method, and equipment are described as follows.

### 3.1. Preparation of Specimen

The GP specimen preparation procedures were as follows: (1) Premix GGBFS and MgO powders in a mixer. (2) Pour activator into the premixed powder and stir it until the slurry became uniform. (3) Cast the above slurry into mold and seal. (4) Set aside the specimens at room temperature. (5) Unmold and measure the initial length of the specimen. (6) Cure the specimen under four environments.

### 3.2. Experimental Planning

This study divided the GP specimen into two groups. The first group fixed A/B as 0.53 ± 0.03, and another group fixed the fluidity as 110 ± 5% of the flow table test. In addition, this study also investigated the effect of unmolding time on the length change ratio. Thus, part of the specimen was unmolded at the age of 3 h and the other was 24 h. The specimens unmolded at the age of 3 h were activated with 2 M, 4 M, and 6 M molar activators; and the specimen unmolded at the age of 24 h were activated with 4 M, 6 M, and 8 M molar activators. The addition of MgO was 0%, 10%, 15%, and 20% in weight of GGBFS. The environmental factor to the length change ratio of GP is also considered. The GP specimen is cured in four environments, i.e., room temperature, air-dry, water bath (23 °C), or steam (65 °C). Each combination of factors prepared three specimens and the total number of specimens is 180, as shown in Table 3.

### 3.3. Experimental Method and Equipment

The fluidity of the slurry was tested according to ASTM C230 standards. The length change ratio referred to ASTM C157 standards of curing in the air-dry environment using a temperature and humidity chamber. In addition, the specimens were also cured in the water tank or steam chamber. XRD was used to detect the hydration reaction of MgO. If there is a peak of Mg(OH)_2_, it means that MgO is hydrated and has expansion behavior.

## 4. Result and Discussion

The test results and discussion of fluidity, length change ratio, and XRD analysis are as follows.

### 4.1. Slurry Fluidity

**Figure 1:**
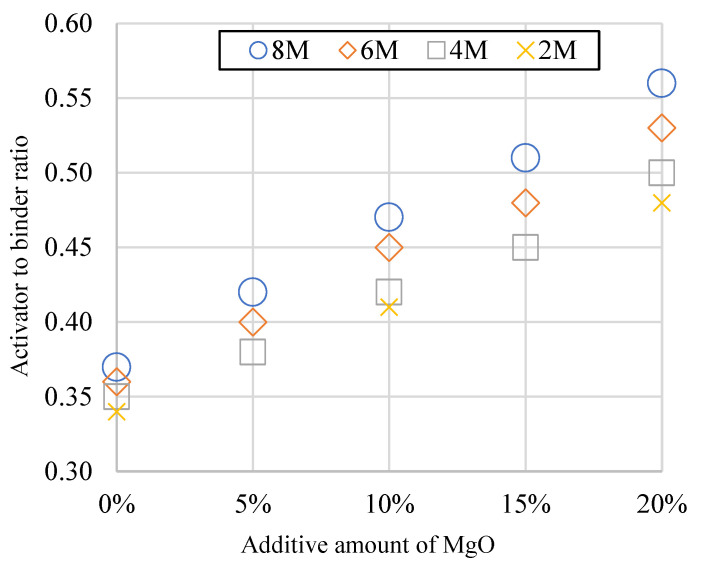
The activator to binder ratios of GP contains different amounts of MgO to make the fluidity of 110 ± 5%.

The fluidity of the fresh slurry was decided by the average value of the measured slump length in four directions, 0, 45, 90, and 135 degrees after the vibration of the flow table. When the fluidity was fixed under 110 ± 5%, the corresponding values of A/B are shown in Figure 1. The result shows that to maintain the workability of the GP slurry, the A/B was increased with the addition of MgO and the molarity of the activator due to the absorption of water by addition powder. Besides, the viscosity was increased with the molarity, which presented the same trend as the literature [55,56,57].

### 4.2. Length Change Ratio

**Figure 2:**
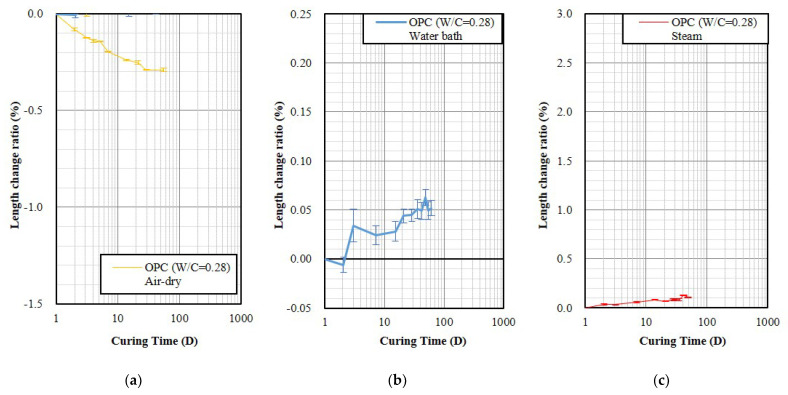
The length change ratios of the OPC specimens demold at age of 24 h and then cured in 3 environments. (**a**) Air-dry; (**b**) Water bath; (**c**) Steam.

First, the shrinkage characteristics of OPC will be described to compare the different shrinkage of GP and OPC. The water-cement ratio (W/C) of the OPC specimen was 0.28, which is sufficient to produce a fluidity of 110 ± 5%. The OPC was unmolded after 24 h and the length change ratio was −0.291%, 0.045%, and 0.088% under air-dry, water bath, and steam curing conditions until 28 days of age, respectively. Figure 2 shows that the length change ratio of the OPC specimens will slightly expand when cured in water and steam, indicating that moisture can prevent shrinkage. This result is consistent with the literature [58,59,60]. The OPC cured in the air-dry environment exhibits shrinkage less than 0.3%.

**Figure 3:**
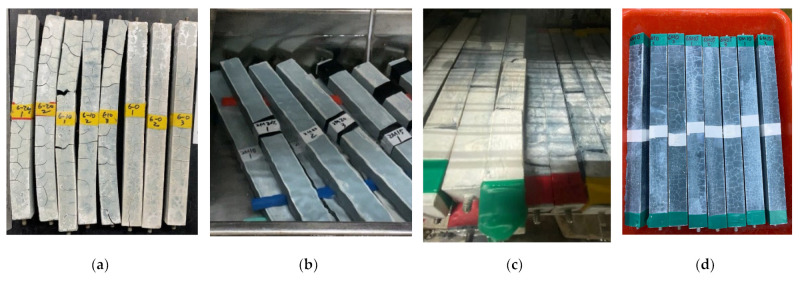
The appearance of GP specimen cured in three environments at the age of 7 days. (**a**) Air-dry; (**b**) Water bath; (**c**) Steam; (**d**) Room temperature.

Figure 3 shows the appearance of GP specimens after seven days in four curing environments. In addition to the water curing, the other three types of curing show great length variation. Room temperature shows obvious cracks. For air-dry curing, parts of specimens were bent due to large shrinkage. For water bath curing, there is no obvious change in appearance. For steam curing, the specimens ruptured into 2–4 pieces without bending, which may be due to the expansion. To measure the length of those fractured specimens, the fractured pieces were temporarily connected as possible during the measurement.

**Figure 4:**
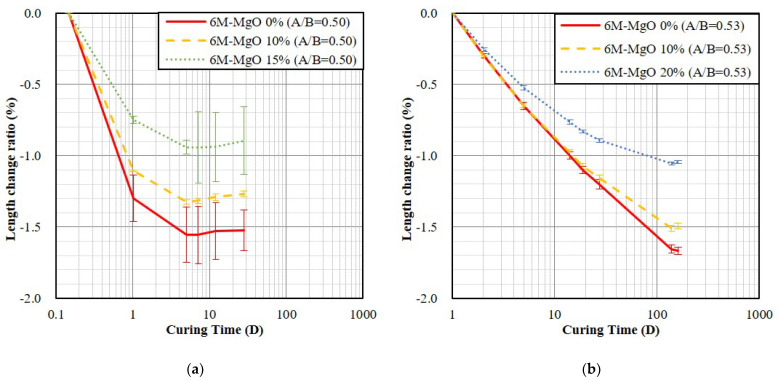
The length change ratios of the GP specimens (fixed A/B). (**a**) 3 h unmolding and A/B is 0.50 (room-temperature); (**b**) 24 h unmolding and A/B is 0.53 (air-dry).

The composition, curing conditions, A/B ratio, fluidity, and the 28-day length change ratio of all the GP specimens unmold at the age of 3 h and 24 h are listed in Table 4 and Table 5, respectively. In the fixed A/B and room temperature curing conditions, the length change ratio of the specimens with unmolding time at the age of 3 h was increased from −1.522% to −0.895% by increasing the addition of MgO from 0% to 15%, respectively; the shrinkage was reduced by 41.2%, as shown in Figure 4a. In the fixed A/B and air-dry curing conditions, the length change ratio of the specimens with unmolding time at the age of 24 h by increasing the addition of MgO from 0% to 20%, the shrinkage of GP specimens could be reduced by 25.6%, as shown in Figure 4b. The result indicated that during the fixed A/B condition, the specimens containing more MgO exhibited less shrinkage. The reason might be the hydration of MgO, which form the Mg(OH)_2_ and increase its volume [43].

In the fixed fluidity and air-dry curing conditions, the result shows that the length change ratio of 4 M without MgO specimens with the unmolding time at the age of 3 h and 24 h are −2.642% and −0.752%, respectively; and the length change ratio of 6 M specimens are −1.945% and −0.728%, respectively, which indicates that the earlier unmolding time exhibits the larger shrinkage. Besides, with increasing MgO content, the A/B and shrinkage of GP are increased.

**Figure 5:**
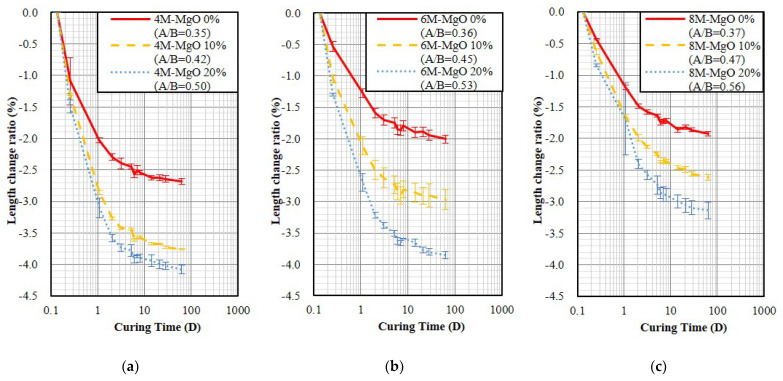
The length change ratios of the GP specimens fixed fluidity and demold at age of 3 h and then air-dry curing. (**a**) 4 M; (**b**) 6 M; (**c**) 8 M.

**Figure 6:**
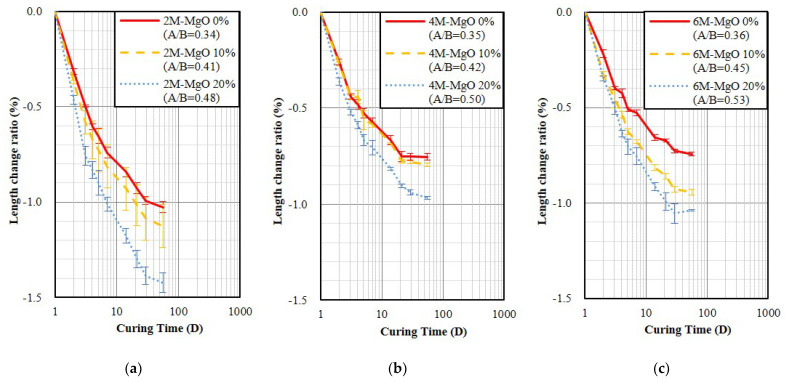
The length change ratios of the GP specimens fixed fluidity and demold at age of 24 h and then air-dry curing. (**a**) 2 M; (**b**) 4 M; (**c**) 6 M.

Figure 5 and Figure 6 show the air-dry curing result of the specimen fixed fluidity and unmold at age of 3 and 24 h, respectively. The results indicate that the addition of MgO increased shrinkage, showing an opposite trend from the set fixed A/B. In addition, the results also show the trend of shrinkage decreased slightly with increasing activator molarity from 2 M to 8 M, which is consistent with the literature, increasing the NaOH molarity of the activator can reduce GP shrinkage [42]. It was also found that earlier unmolding resulted in larger shrinkage. Compared to the GP specimens without containing MgO with the OPC specimen, shown in Figure 2a and Figure 6, the 28-day shrinkage of GP specimens cured in the air-dry environment was 2.4–3.3 times larger than OPC.

**Figure 7:**
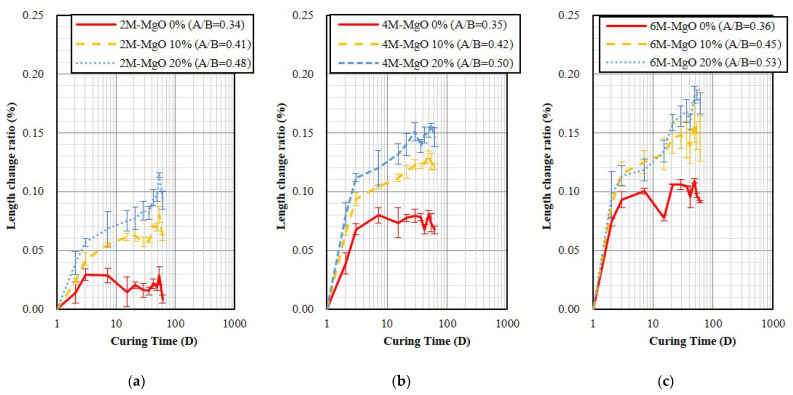
The length change ratios of the specimens demold at age of 24 h and then water bath curing. (**a**) 2 M; (**b**) 4 M; (**c**) 6 M.

**Figure 8:**
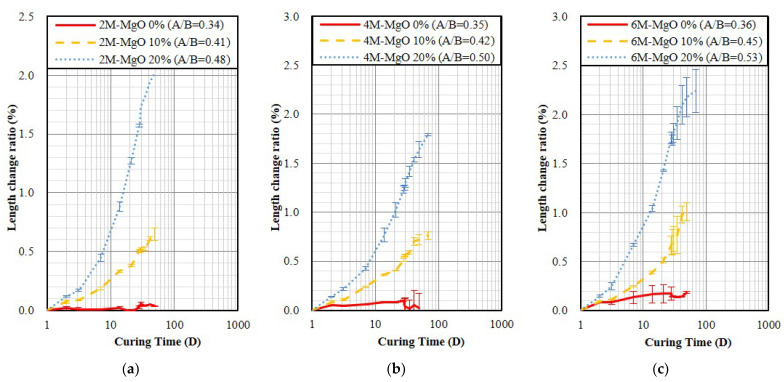
The length change ratio of the specimen demold at age of 24 h and steam curing. (**a**) 2 M; (**b**) 4 M; (**c**) 6 M.

Figure 7 shows the water bath curing result, it indicates that the GP specimen will slightly expand. It was also found that the expansion will increase with a higher NaOH molarity of activator and MgO content. In addition, compared with OPC, as shown in Figure 2b, all GP specimens were 1.26–3.6 times larger than OPC, besides the 2 M specimen without containing MgO. Figure 8 shows the steam curing result, which indicates that the GP specimen will expand, and the more MgO contents, the more rapid the expansion will be. Both GP specimens without containing MgO and OPC specimens exhibited similar expansion, as shown in Figure 2c. Furthermore, as the NaOH molarity of the activator increases from 2 M to 6 M, the expansion also tends to increase slightly.

### 4.3. XRD Analysis

**Figure 9:**
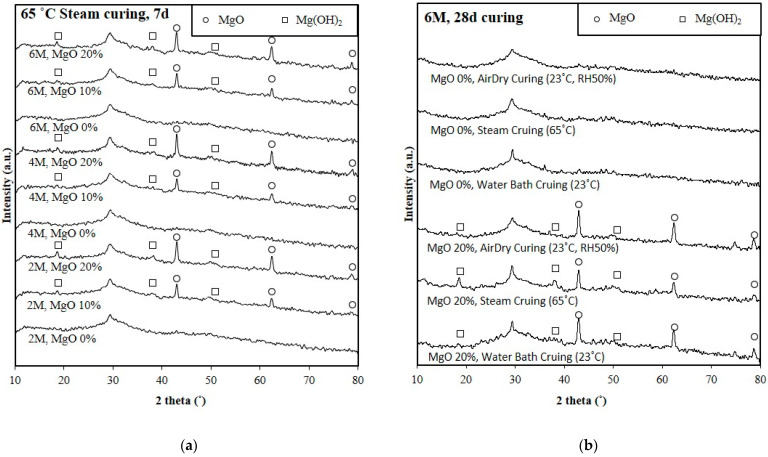
XRD analysis of the GGBFS-based GP specimens contains different amounts of MgO and various curing environments. (**a**) Steam curing for 7 days; (**b**) 6 M GP curing in 3 environments for 28 days.

XRD was used to check the Mg(OH)_2_ that occurred from the MgO hydration. When there was Mg(OH)_2_, the volume of MgO was expanded with hydration, and the shrinkage of GP specimen might be compensated. Figure 9a is the XRD analysis result of the 2 M to 6 M GP specimens cured in the steam environment for seven days. The results show that all specimens with the addition of MgO had characteristic peaks of MgO and Mg(OH)_2_. With the addition of MgO from 10% to 20%, the diffraction intensities of MgO and Mg(OH)_2_ tended to increase. Figure 9b presents the XRD analysis result of the 6 M GP specimens cured in different environments for 28 days. The results show that the peak width of around 30 degrees tends to narrow when the specimens were cured in high humidity and temperature. The specimens with the addition of 20% MgO and cured in the air-drying environment did not have obvious characteristic peaks of Mg(OH)_2_, which indicated the hydration of MgO did not occur. The specimens with the addition of 20% MgO and cured in the water bath environment show very weak characteristic peaks of Mg(OH)_2_, which is represented that only a few MgO hydrated to Mg(OH)_2_. Although the steam-cured specimens show part of the MgO hydrated to Mg(OH)_2_, there remain strong characteristic peaks of MgO, which is represented that the specimens will continue to expand after 28 days of curing. The length change ratio test also conforms to it, as seen in Figure 8. In a dry environment, Mg(OH)_2_ could not be found in the GP specimen containing MgO, which indicated the MgO did not compensate for the shrinkage; yet in the high temperature and humidity environment, the GP containing MgO would overexpand.

## 5. Machine Learning for Main Factors of Length Change Ratio

**Figure 10:**
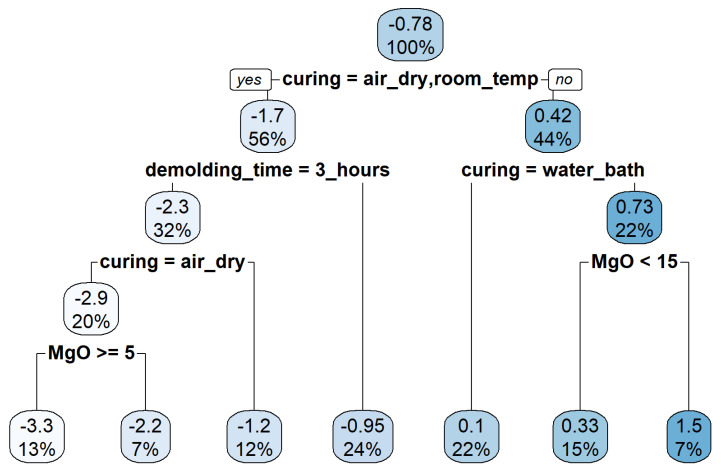
The regression tree for the rate of length change using data of 124 specimens.

We aggregated all the specimens (124 records) for a combined analysis to offer a comprehensive picture of the length change ratio of the specimens examined under the various conditions stated in the preceding section. The goal of this analysis was to ascertain the most significant factors affecting the length change ratio after 28 days. This analysis utilized three statistical and machine learning methods: decision tree, multiple regression, and random forest. The decision tree in this analysis was implemented using the R package rpart(). The produced decision tree, which is a regression tree because it produces numerical values, is shown in Figure 10.

As illustrated in Figure 10, the regression tree analysis yields the following rules:

1. If the specimen is air-dry cured, unmolded after three hours, and the MgO amount is more than or equal to 5%, then the expected length change ratio is −3.3%.
2. If the specimen is air-dry cured, unmolded after three hours, and the MgO amount is less than 5%, then the expected length change ratio is −2.2%.
3. If the specimen is room-temperature cured and demolded three hours later, then the expected length change ratio is −1.2%.
4. If the specimen is air-dry or room-temperature cured and demolded 24 h later, then the expected length change ratio is −0.95%
5. If the specimen is water-bath cured, then the expected length change ratio is 0.1%.
6. If the specimen is steam cured and the MgO amount is less than 15%, then the expected length change ratio is 0.33%.
7. If the specimen is steam cured and the MgO amount is more than or equal to 15%, then the expected length change ratio is 1.5%.

These rules and Figure 10 demonstrate unequivocally that the curing condition is the single most critical factor affecting the length change ratio of the specimens after 28 days. The unmolding time or MgO amount comes after the curing condition. In rules 1 and 2, increased MgO results in increased shrinkage. However, in rules 6 and 7, an increase in MgO causes an increase in expansion. This is consistent with the observation made in the preceding section.

**Figure 11:**
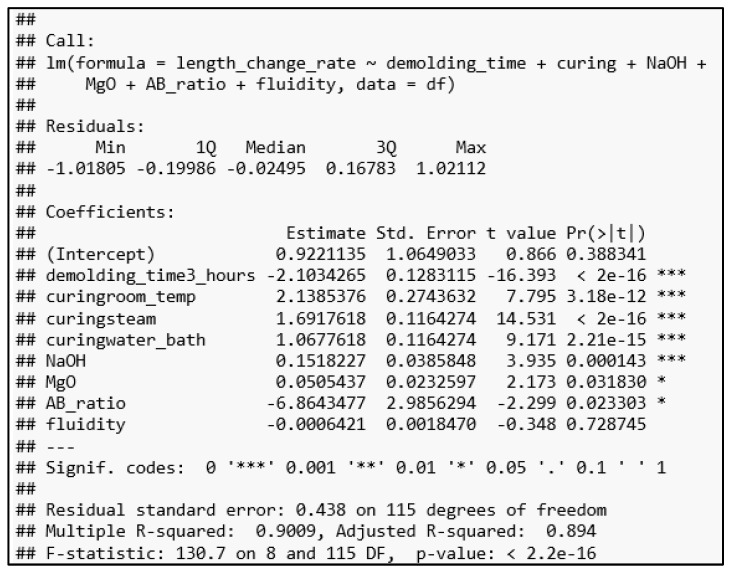
The multiple regression analysis for the length change ratio basing on 124 specimens’ data.

The lm() function in R was used to perform a multiple regression analysis. The outcome is depicted in Figure 11. When lm() came across the two-level factor variable, unmolding time, it created a new binary variable (unmolding_time3_hours). This variable (unmolding_time3_hours) was set to 1 if the specimen was unmolded after three hours and to 0 if it was unmolded after 24 h. Similarly, lm() established three binary variables (curingroom_temp, curingsteam, and curingwater_bath) for the curing condition. Their values were set to 0 or 1 in accordance with one of the four curing conditions (air-dry, room-temperature, water-bath, and steam).

As illustrated in Figure 11, the multiple regression model is statistically significant at a *p*-value of less than 2.2 × 10^−16^. Additionally, the variables relating to the unmolding time and curing condition, as well as the NaOH concentration, are all statistically significant at the 0.001 level. The MgO amount and A/B ratio are also significant, but only at the 0.05 level. It is worth noting that MgO amount has a coefficient of 0.05, indicating that it has the expansive effect described in the literature. The shrinkage impact is primarily caused by the A/B ratio (−6.86) and the three-hour unmolding (−2.10). As a result, these two variables compensate for and overshadow MgO’s expansive effect.

**Figure 12:**
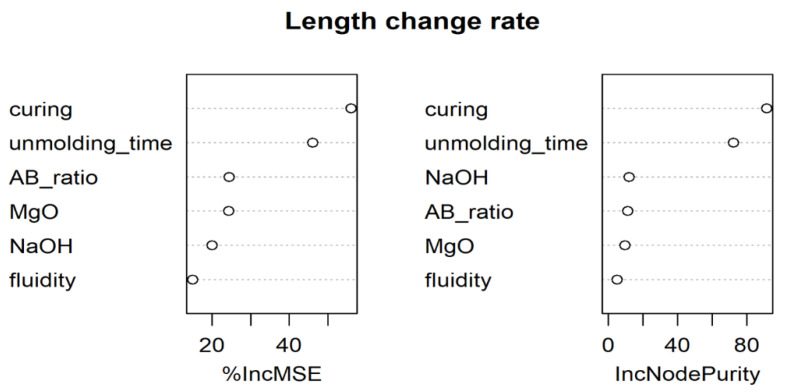
The most significant influencing factors on the length change ratio as determined by random forest.

Finally, we utilized the R package “randomForest” to assess the specimens’ length change rate with the average of 1000 individual decision trees and to determine the most significant factors. The outcome is depicted in Figure 12. The two most critical factors are the curing condition and unmolding time, as measured by both the mean decrease accuracy (%IncMSE) and the mean decrease Gini (IncNodePurity). This result is consistent with those of decision tree and multiple regression analysis. In comparison to the curing condition and unmolding time, the other four factors are far less critical. Among these, the MgO amount is only the third or fifth most important factor in influencing the rate at which the specimens change length.

## 6. Conclusions

In this study, we utilized the addition of MgO to compensate for the shrinkage of the GGBFS-based GP cured in different environments and concluded the following from the experimental and analytical results:

1. When the activator to binder ratio was fixed, the shrinkage of the GP cured in an air-dry and room temperature environment was decreased with the addition of MgO.
2. The workability of fresh GP slurry will reduce with the addition of MgO. It is necessary to increase the activator to binder ratio to maintain fluidity. However, the shrinkage of GP will increase.
3. Comparing GP specimens unmolded at 3 h and 24 h, delaying the demolding time can reduce shrinkage.
4. The XRD results indicate the shrinkage of GP cured in the air-dry environment cannot be compensated by MgO because there is not enough water to hydrate the MgO to form Mg(OH)_2_.
5. The GP specimens cured in a moist environment (water-bath and steam) can avoid shrinkage and produce slight expansion, while the addition of MgO may cause over-expansion and fracture.
6. Increasing the NaOH molarity of the activator from 2 M to 8 M can reduce the shrinkage of GP under air-dry curing, and can also increase the expansion under water bath can steam curing.
7. The results of the statistical and machine learning analysis show that the length change ratio of GP is mainly affected by curing conditions and unmolding time, while the addition of MgO is not the main factor.

## Figures and Tables

**Table 1:** The chemical composition of GGBFS.

**Composition**	CaO	SiO_2_	Al_2_O_3_	MgO	SO_2_	TiO_2_	Fe_2_O_3_	MnO_2_	K_2_O	P_2_O_5_	Cr_2_O_3_	SrO	ZrO_2_	Total
**Percentage** (%)	47.32	32.43	9.96	5.16	2.29	0.80	0.60	0.55	0.41	0.29	0.02	0.11	0.06	100.00

**Table 2:** The chemical composition of magnesium oxide.

**Composition**	MgO	SiO_2_	SO_2_	CaO	K_2_O	P_2_O_5_	TiO_2_	MnO_2_	Total
**Percentage** (%)	94.67	2.22	1.52	0.89	0.34	0.27	0.05	0.03	100.00

**Table 3:** The experimental planning of the length change ratio.

Unmolding Time	Group	Curing Environment	NaOH Molarity (M)	MgO (%)	Number of Specimen
3 h	Fix A/B	Room Temp.	6	0, 10, 15	9
	Fix Fluidity	Air-Dry	4, 6, 8	0, 10, 20	27
		Steam	4, 6, 8	0, 10, 20	27
		Water bath	4, 6, 8	0, 10, 20	27
24 h	Fix A/B	Air-Dry	6	0, 10, 20	9
	Fix Fluidity	Air-Dry	2, 4, 6	0, 10, 20	27
		Steam	2, 4, 6	0, 10, 20	27
		Water bath	2, 4, 6	0, 10, 20	27

**Table 4:** The length change ratios of the specimens unmolded at the age of 3 h.

Fixed	Curing Environment	NaOH Molarity	MgO (%)	A/B	Fluidity (%)	Length Change Ratio ^1^ (%)	Average of Length Change Ratio (%)
A/B	Room Temperature	6 M	0	0.50	>200 ^2^	−1.521, −1.380, −1.666	−1.522
			10		155	−1.244, −1.236, −1.325	−1.268
			15		98	−1.143, −0.670, −0.873	−0.895
Fluidity	Air-Dry	4 M	0	0.35	110 ± 5	−2.698, −2.606, −2.623	−2.642
			10	0.42		−3.713, −3.739	−3.726
			20	0.50		−4.084, −3.972, −4.006	−4.021
		6 M	0	0.36		−2.014, −1.956, −1.864	−1.945
			10	0.45		−2.758, −2.841, −3.111	−2.903
			20	0.53		−3.768, −3.834	−3.801
		8 M	0	0.37		−1.900, −1.854, −1.841	−1.865
			10	0.47		−2.599, −2.558, −2.525	−2.561
			20	0.56		−3.209, −2.993, −3.082	−3.095

^1^ The result was obtained by the age of 28 days compared to the age of 3 h. ^2^ Test limitation of the flow table test is 200%.

**Table 5:** The length change ratios of the specimens unmolded at the age of 24 h.

Fixed	Curing Environment	NaOH Molarity	MgO (%)	A/B	Fluidity (%)	Length Change Ratio ^1^ (%)	Average of Length Change Ratio (%)
A/B	Air-Dry	6 M	0	0.53	>200 ^2^	−1.222, −1.165, −1.217	−1.201
			10		168	−1.146, −1.181, −1.142	−1.156
			20		113	−0.906, −0.895, −0.879	+0.893
Fluidity	Air-Dry	2 M	0	0.34	110 ± 5	−1.015, −0.987, −0.976	−0.993
			10	0.41		−1.140, −0.959, −1.162	−1.087
			20	0.48		−1.342, −1.430, −1.389	−1.387
		4 M	0	0.35		−0.741, −0.744, −0.772	−0.752
			10	0.42		−0.779, −0.790, −0.778	−0.782
			20	0.50		−0.946, −0.948, −0.925	−0.940
		6 M	0	0.36		−0.723, −0.740, −0.721	−0.728
			10	0.45		−0.944, −0.913, −0.930	−0.929
			20	0.53		−1.030, −1.022, −1.112	−1.055
	Steam	2 M	0	0.34		+0.028, +0.036, +0.011	+0.025
			10	0.41		+0.491, +0.498, +0.512	+0.500
			20	0.48		+1.557, +1.584, +1.571	+1.571
		4 M	0	0.35		+0.098, +0.101, +0.090	+0.096
			10	0.42		+0.501, +0.531, +0.510	+0.514
			20	0.50		+1.192, +1.269, +1.238	+1.233
		6 M	0	0.36		+0.254, +0.132, +0.141	+0.176
			10	0.45		+0.688, +0.744, +0.564	+0.665
			20	0.53		+1.698, +1.781, +1.814	+1.764
	Water bath	2 M	0	0.34		+0.023, +0.013, +0.013	+0.016
			10	0.41		+0.058, +0.053, +0.061	+0.057
			20	0.48		+0.088, +0.075, +0.089	+0.084
		4 M	0	0.35		+0.076, +0.076, +0.086	+0.079
			10	0.42		+0.113, +0.125, +0.131	+0.123
			20	0.50		+0.135, +0.150, +0.168	+0.151
		6 M	0	0.36		+0.104, +0.102, +0.111	+0.106
			10	0.45		+0.161, +0.139, +0.144	+0.148
			20	0.53		+0.154, +0.171, +0.167	+0.164

^1^ The result was obtained by the age of 28 days compared to the age of 24 h. ^2^ Test limitation of the flow table test is 200%.

## Data Availability

Not applicable.
